# The Role of Reticulate Evolution in Creating Innovation and Complexity

**DOI:** 10.1155/2012/418964

**Published:** 2012-07-12

**Authors:** Kristen S. Swithers, Shannon M. Soucy, J. Peter Gogarten

**Affiliations:** Department of Molecular and Cell Biology, University of Connecticut, Storrs, CT 06269-3125, USA

## Abstract

Reticulate evolution encompasses processes that conflict with traditional Tree of Life efforts. These processes, horizontal gene transfer (HGT), gene and whole-genome duplications through allopolyploidization, are some of the main driving forces for generating innovation and complexity. HGT has a profound impact on prokaryotic and eukaryotic evolution. HGTs can lead to the invention of new metabolic pathways and the expansion and enhancement of previously existing pathways. It allows for organismal adaptation into new ecological niches and new host ranges. Although many HGTs appear to be selected for because they provide some benefit to their recipient lineage, other HGTs may be maintained by chance through random genetic drift. Moreover, some HGTs that may initially seem parasitic in nature can cause complexity to arise through pathways of neutral evolution. Another mechanism for generating innovation and complexity, occurring more frequently in eukaryotes than in prokaryotes, is gene and genome duplications, which often occur through allopolyploidizations. We discuss how these different evolutionary processes contribute to generating innovation and complexity.

## 1. Introduction

Reconstruction of the Tree of Life attempts to represent the organismal histories of all of life on earth on a single bifurcating tree. Since the dawn of the molecular age, and, more so recently, with the numerous whole-genome sequences that are now available, it has become apparent that reticulate evolutionary processes such as horizontal gene transfer (HGT), genome fusion, and incomplete lineage sorting have a profound impact on microbial and eukaryotic evolution. These processes dissolve or embed the lines of vertical descent that are a hallmark of the tree of life into net-like relationships between genomes and organisms. To more accurately describe the complexity of organismal histories many groups have proposed net-like reconstructions of life's history [1] to account for the lines of vertical descent and lateral lines created from reticulate processes; the “rooted net of life” [2], the “forest of life” [3, 4], and the “rhizome of life” [5, 6] are a few examples.

HGT is the nonvertical transmission of genetic material, that is, the exchange of genetic information between organisms not in an ancestor descendant relationship. HGT causes individual genes in a genome to have vastly different evolutionary histories. Studies show HGT occurs more frequently between closely related organisms than in divergent organisms [7, 8]. Closely related organisms tend to have similar sequences and intracellular environments. These similarities allow for more opportunity for homologous recombination and for an easier integration of the transferred gene into the metabolic and regulatory networks of the recipient. However, there are increasing examples of HGTs between divergent species, even across domain boundaries, revealing that barriers to HGT can occasionally be overcome. Examples include the highways of HGTs [9] that exist between divergent organisms: members of the Thermotogae phylum share about half of their genes with both the Firmicutes and the Archaea [10], and the Aquificae share many genes with the Epsilonproteobacteria [11]. Many of these successful HGTs allow for innovations in metabolism and body plan that provide a selective advantage to the organisms involved and allow expansion into new ecological niches.

Transferred genes can be distinguished based on their long- and short-term impact on the fitness of the recipient (Table 1). Genes that provide an adaptation create a selective advantage for the recipient and have a higher chance to persist over longer periods of time. As their frequency in the population increases over time these genes will become fixed. Examples of these “beneficial” HGTs are those that allow the recipient to expand into a previously empty ecological niche. These provide a huge increase in fitness to the recipient, even if the transferred gene has not yet adapted perfectly to the genomic and regulatory environment of the recipient [12]. Many of the genes that extend, enhance, or create new metabolic pathways fall into this category. These genes may be selfish in Dawkins' [13] original definition, but they cooperate with the other genes in the organism's genome and provide a selective advantage for the organism.

Many other, and possibly most, transferred genes that can be identified in the pan-genome [16] of bacterial or archaeal populations may be selectively neutral or nearly neutral to their carriers [14]. Many of these genes will be lost after a few generations; however, a few may be fixed through random genetic drift. It could be argued that most of the endosymbiant *Wolbachia* to host transfers are selectively neutral or nearly neutral. Almost all of the *Wolbachia* genes are found in the host genome and their transcript levels are very low [17]. This low transcript level may indicate that these genes do not provide a function to the host and supports the notion that many genes transferred from the symbiont are only transiently present in the host nuclear genome. Although the majority of these transferred genes are transcribed at very low level, two hypothetical proteins in the *Aedyes aegypti* originating from *Wolbachia* have been maintained in the nuclear genome for a long period of time and are transcribed at higher levels than background suggesting these genes were fixed in the population [18].

Some transferred genes initially are like infections in that their survival and spread is through a mechanism that decouples the genes propagation from host replication and host fitness. Although the propagation of these selfish genetic elements is decoupled from the host's genetic machinery, the element does utilize the host's resources to propagate through a population. In this sense these genetic elements can be considered parasitic. To more clearly distinguish them from the selfish gene concept in Dawkins' gene-centered view of evolution, which considers all genes as selfish, we term these elements as parasitic genetic elements and their transfers “parasitic HGTs”; examples include inteins and self-splicing introns. Initially, a self-splicing molecular parasite may provide little or no advantage to the host but may later adapt a function to benefit the host. Many inteins and group I introns contain a homing endonuclease (HE) that provides mobility to the element and allows them to follow a life cycle known as the homing cycle [19]. Briefly, the homing cycle begins when an allele with an HE is horizontally transferred to a recipient in a new population or species that before the invasion harbored only alleles without HE [20]. Through faster than Mendalian inheritance the HE containing parasite spreads through the population, leaving little or no detrimental effects on the host. However, once all the members of the population have the HE containing element the HE containing genetic element starts to degrade. To escape this cycle, over time the parasites may adapt to provide a beneficial function for the host [7] or are maintained through neutral pathways to complexity as discussed below for the case of the *dnaE* intein [21, 22].

Transferred genes can be integrated into the recipient genome by homologous recombination or through illegitimate recombination [23]. The former process requires stretches of similar sequences; however, the stringency of this requirement depends on the activity of the mismatch repair system [24]. The similarities necessary for homologous recombination can be due to the presence of a homolog in the recipient genome or can be created through transposable elements present in the recipient that jump into the transferred extrachromosomal genetic material [25]. Transferred DNA also can be integrated independent of sequence similarity through double-strand break repair pathways, such as nonhomologous end joining, allowing for the integration of DNA from divergent organisms [7]. Transposable elements can also facilitate transfer and integration into recipient DNA. One such example is the integrative and conjugative elements (ICEs). ICEs have been implicated in transfer of genes involved in antibiotic and heavy metal resistance, nitrogen fixation, virulence, biofilm formation, and the degradation of aromatic compounds (for reviews see [26, 27] and references therein), providing another example for multiple levels of selection, in this case benefiting both the transferred genes and the recipient.

Although HGT appears to be more prevalent in prokaryotes, more and more examples of HGT are being documented in single-celled and even multicellular eukaryotes (see [28] and below for examples of transfer from bacteria to eukaryotes). Related driving forces in creating innovation and complexity in eukaryotic lineages are gene and whole genome duplications. Genome fusion resulting from hybridization between members of related species, a frequent pathway towards polyploidization, is akin to HGT in that it results in mosaic genomes and that the resulting gene family expansion is due to reticulate evolution. Observed in plants [29], animals [30, 31], and fungi [32, 33] whole-genome duplication followed by neofunctionalization and/or subfunctionalizations have been implicated in providing the building blocks for more complex developmental and metabolic pathways.

Gene, genome duplication, and HGT, regardless of the type of selection, beneficial, neutral, or parasitic, are all reticulate processes that affect evolution across all domains of life. Here we explore how the process of HGTs can expand metabolic pathways, allow for microorganisms to adapt to new host ranges, expand environmental niches, and even influence multicellular eukaryotes. We also explore how “parasitic HGTs” can ultimately lead to innovation and increased complexity. Additionally, we discuss how gene and whole-genome duplications can give rise to novel pathways that are important for development.

## 2. HGT and Expansion Metabolic Pathways

HGTs can lead to the enhancement, expansion, and construction of more complex metabolic pathways. About two-thirds of the annual biogenic methane is produced from the acetoclastic methanogenesis pathway, which is exclusively carried out by the methanogenic eurarchaeal order Methanosarcinales [34]. Most members of this group carry out the conversion of acetate to acetyl-coenzyme A using the acetyl-CoA synthesis pathway. However, members of the more widely distributed *Methanosarcina* use a variation on this pathway, which uses the enzymes acetate kinase (AckA) and phosphoacetyl treasferase (Pta) [34]. Both the *ackA* and *pta* genes were shown through multiple phylogenetic methods to be transferred in one event from the cellulolytic clostridia, where the encoded enzymes are used to produce acetate as a product of fermentation, to *Methanosarcina* [35], where the same enzymes are used to produce acetyl-CoA.

Another example of an expanded pathway created by HGT is found in the Thermotogae phylum. Some of the lower-temperature lineages are able to produce vitamin B_12_ using the cobinamide salvage pathway [36] (Figure 2). In this pathway a partial B_12_ molecule is scavenged from the environment and subsequently modified to produce an active B_12_ molecule. This method of B_12_ production was shown to be the ancestral pathway for the Thermotogae lineage by presence and absence of the genes in the phylum (Figure 2). A later HGT allowed the *Thermosipho* genus to synthesize B_12_ *de novo* from glutamate, through transfer of twenty-one genes from the Firmicutes.

An enhancement of a pathway is observed in HGT events between eukaryotic species of grasses. Some members of the *Alloteropsis *grasses have acquired highly functional genes for C_4_ photosynthesis from the Cenchrinae and Melinidinae: phosphoenolpyruvate carboxylases (ppc) were likely transferred from both the Cenchrinae and Melinidinae, and phosphoenolpyruvate carboxykinase (pck) was transferred from the Cenchrinae. Christin et al. hypothesize that before the arrival of these genes the *Alloteropsis* may have had a subfunctional C_4_ CO_2_-fixation pathway, as in the case of the extant *A. semialata* subsp. *semialata* grass, which did not receive these HGTs. This enhancement of the C_4_ pathways allows for adaptation of the grass to warm and arid climates [37].

The metabolic pathways expanded and enhanced through HGT allow for an occupation of a new ecological niche. The *Thermosipho* can now produce B_12_ and thrive in an environment where no partial B_12_ derivatives are present, while members of the genus *Methanosarcina* are able to produce most of the world's methane from acetate and the *Alloteropsis *grasses can thrive in warm and arid climates.

## 3. HGT and Metabolic Innovations

**Figure 1:**
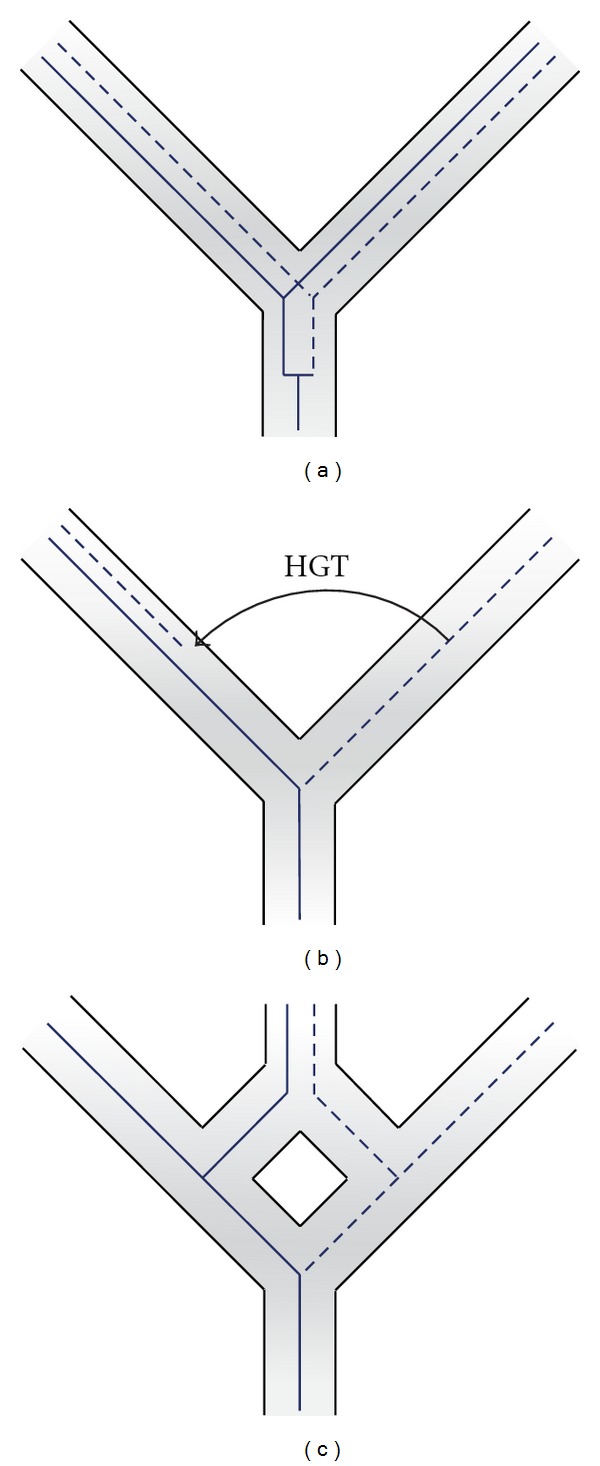
Types of genetic duplications. (a) Shows an autochthonous duplication, which can happen either through tandem duplication, segmental duplication, chromosomal duplication, genome duplications, or retro-transposition. (b) Shows gene family expansion through HGT. Following the divergence of two lineages orthologous genes diverge in sequence and possibly in function. These orthologs can be brought together in a single genome through HGT or allopolyploidization (c). The scenarios depicted in (c) and (b) explain an apparent duplication through reticulated evolution.

**Figure 2:**
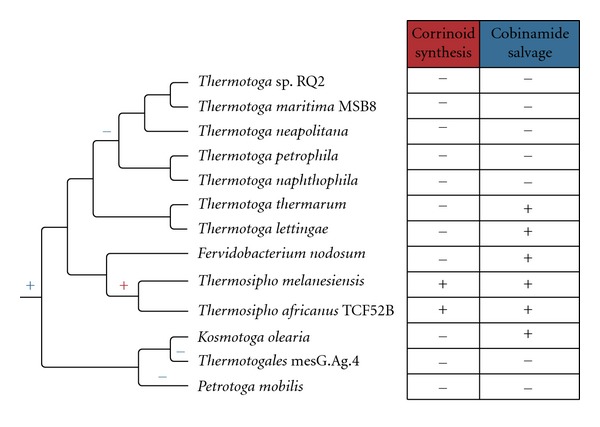
Distribution of the two gene clusters involved in vitamin B_12_ biosynthesis among the Thermotogae phylum. The corrinoid synthesis gene cluster contains genes for the first part of the *de novo* B_12_ synthesis pathway and the cobinamide salvage gene cluster contains genes that synthesize vitamin B_12_ from cobinamides, incomplete B_12_ molecules. Together these two gene clusters complete the *de novo* B_12_ biosynthesis pathway. Presence of a gene cluster is denoted by (+) and absence is denoted by (−). The most parsimonious explanation for the extant presence/absence patterning for the cobinamide salvage gene cluster is one gain at the root of the phylum and three losses marked by blue and (+) and (−) and for the corrinoid synthesis gene cluster one gain marked by a red (+). This suggests the cobinamide salvage pathway was present in the ancestor of the Thermotogae phylum and the genes for complete *de novo* synthesis were gained in a later event by the *Thermosipho* lineage.

Members of at least six different bacterial phyla use chlorophyll-based photosynthesis to gain energy from light [38, 39]. Comparative phylogenetic analysis revealed that horizontal gene transfer played an important role in evolution and distribution of bacterial photosynthesis [40, 41]. The assembly of the electron transport chain that allows the use of water as electron donor likely represents the gene transfer event that most changed Earth's biosphere [42, 43]. Chloroflexi (green filamentous bacteria) and purple bacteria possess a photosynthetic reaction center similar to photosystem II of the cyanobacteria; whereas the reaction centers in Chlorobi (green sulfur bacteria) and Heliobacteria (Firmicutes) are similar to the photosynthetic reaction center I in cyanobacteria [39, 44]. However, in the cyanobacteria photosystem I and photosystem II are present, and only when the two divergent types of reaction centers work in series do the harvested photons provide sufficient energy to lift electrons over the electrochemical potential difference between water and NADP. It is theoretically possible that photosystems I and II arose through a within-lineage gene duplication, diverged within the cyanobacteria, and subsequently individual photosystems were transferred to other bacteria. A more likely scenario is that the two photosystems diverged from an ancestral photosystem in diverging lineages (Figure 1(b)), which each used a single photosystem, and that the two distinct photosystems were brought together in the cyanobacterial ancestor through HGT.

The recently described methylaspartate cycle in Haloarchaea [45] provides another example for the creative power of HGT. This cycle provides an alternative to the glyoxylate cycle and the ethylmalonyl-CoA pathway for acetyl CoA to enter central carbon metabolism to synthesize cellular building blocks. According to analyses reported in [45] the key enzymes of the methylaspartate cycle were acquired by the Haloarchaea through gene transfer from different bacteria. Furthermore, before the transfer, these enzymes were part of different pathways in the donor organisms, such as propionate assimilation or glutamate fermentation. The methylaspartate cycle thus represents a metabolic patchwork of enzymes acquired from different donors and combining fragments of different pathways into a novel enzymatic cycle.

## 4. HGT and Innovations in Communities

The human microbiome provides an opportunity to understand a complex community of microorganisms and how HGT has facilitated innovation within a large community of microorganisms. Many traits, such as antibiotic resistance, and xenobiotic metabolism observed in the human gut microbiota are a consequence of HGT. One study showed that antibiotic resistance genes can be transferred to the gastrointestinal microbiome from food sources [46]. Volunteers were fed chicken, which had a strain of vancomycin-resistant *Enterococcus faecium*, and vancomycin resistance was transferred to *E. faecium* in the human gut. Other studies in Japanese individuals showed that genes for porphyranases, agarases, and alginases, which facilitate the breakdown of red and brown algae (seaweed) in the human gut, were likely transferred from marine bacteria to Japanese gut symbiont *Bacteroidetes *[47, 48]. These HGTs not only allow the gut bacteria to utilize seaweeds as a novel carbon source, but confer secondary benefits to the human host, which can now utilize seaweed as a nutrient source. The act of introducing foreign material to the gut microbiota (consuming a food source) facilitates interactions between the microbiome and the microorganisms on that food source. This interaction encourages possible HGTs from microorganisms outside the gut and allows for constant innovation and evolution of our microbiome to cope with the frequent changes in the gut environment, reinforcing the “you are what you eat” saying. These findings also confirm that the holobiont (host plus symbiont) can evolve and gain new adaptations without changes in the host's genome, simply by acquiring new symbionts with novel metabolic capabilities [49].

## 5. “Parasitic HGTs” Can Lead to Innovation and Complexity

“Parasitic HGT” involving molecular parasites, such as inteins and group I introns, are HGTs that confer no immediate selective advantage to the host but over time adapt to benefit the host. These inteins and group I introns are self-splicing genetic elements that are made mobile by homing endonucleases, an endonuclease that recognizes target sequences of 12–40 bps [50]. They can evade purifying selection on the organismal level as they cause little or no harm to their host [51]. These HE containing parasites have their own life cycle described by the homing cycle [20, 50, 52]. A possible escape route from this cycle presents itself, if the HE or the intein/intron evolves a beneficial function in the host. One such example of this is found in the mating type switching HO endonuclease in yeast [53]. This endonuclease is left over from what once was a close relative to the large intein in the yeast vacuolar ATPase catalytic subunit, but now facilitates genetic recombination from one mating type to another. This innovation is beneficial to the organism in that it expands the reproductive capabilities of the yeast cell. Another example where an intein may have been retained and adapted to benefit its host is found in bacterial intein-like (BIL) domains. These are degenerated remnants of the HINT domain intein family, which are now thought to function to facilitate rearrangements in hypervariable surface proteins [54, 55]. Over time the HEs of some group I introns are maintained as functional maturases to aid in the folding and splicing of the intron they reside in or other introns that may have lost their self-splicing ability [21, 56]. In these cases parasitic HGTs have facilitated beneficial innovations; however, most of these innovations evolved after a long period of neutral or nearly neutral association between the parasite and host.

Although many “parasitic HGTs” eventually provide some benefit for the host, there are other cases where they are maintained via selectively neutral pathways, which also can lead to higher complexity. The *dnaE* gene, of some cyanobacterial species, is split on two parts (*dnaE1* and *dnaE2*), and each portion has part of an N-terminal or C-terminal intein [57]. An autocatalytic mechanism allows the split inteins to find each other after translation and splice the split protein together, resulting in a functional DNA polymerase III. Deletion or mutation of the intein portions of the split gene results in a nonfunctional DNA polymerase III, a major selective disadvantage for the organism and even possibly detrimental. This intein likely never supplied a selective advantage for the host. Through a series of intermediate steps, each of them neutral or nearly neutral to the organism, a complex processing system emerged that places the intein under strong purifying selection, because the self-splicing reaction of the intein now is necessary to synthesize a functioning DNA polymerase III [22]. The wide distribution of the split intein in *dnaE *in cyanobacteria [58] suggests that this rather complex gene structure is an evolutionarily stable arrangement.

Another mobile genetic element that is frequently transferred and creates novelties and complexity is group II introns. They are thought to be the predecessors of both the eukaryotic spliceosomal introns and non-LTR retrotransposon [59–61]. These self-splicing elements are found in all domains of life; they are made mobile either via retrohoming, using an endonuclease [62], or retrotransposition mechanisms, using a reverse transcriptase [63]. Evidence for group II introns being the ancestors of the spliceosomal intron in eukaryotes includes similar splicing mechanisms, comparable boundary sequences, and secondary structure similarities [64–66]. One hypothesis suggests the group II intron originated in the bacteria and were horizontally transferred from the alphaproteobacterial endosymbiont ancestor of the mitochondria to the genome of the ancestor of the eukaryotic nucleocytoplasm. The presence of introns in most transcripts might have necessitated a separation between transcription and translation, facilitating the emergence of a nucleus [67]. Some of the original introns may have lost their self-splicing activity and relied on other introns and their associated proteins to catalyze the splicing reaction in trans, evolving over time into the spliceosomal machinery. In this scenario, the introns initially proliferated as molecular parasites; however, on the long run they allowed for exon shuffling, alternative splicing, and the nonsense mediated decay pathway to evolve. Interestingly, extant bacterial group II introns maintain self-splicing and mobility, while most mitochondrial and chloroplast group II introns are not mobile and have lost the ability to self-splice. For example, about 20 group II introns present in the organelles of plants have lost their ability to self-splice [68, 69]. However, to maintain functional genes, they must be spliced out thus their maintenance is dependent on the complex interactions with nuclear and plastid splicing factors. Group II introns have also been implicated in genome rearrangements and gene conversion events [70], both of which can cause innovations in gene function and structure.

## 6. Interdomain HGT and Innovation

One of the benefits of HGT is that it can provide a selective advantage for organisms to occupy new niches and expand host ranges. Many interdomain transfers from bacteria to single-celled eukaryotes provided for innovations and adaptation to new environments [28, 71]. In many instances these genes were subsequently transferred between divergent single-cell eukaryotes [28]. One example is the parasitic protozoan *Blastocystis*, which is found in many different animal gut environments and causes gastrointestinal diseases, and has acquired genes for energy metabolism, adhesion, and osmotrophy from various bacterial donors. These transfers have allowed the successful adaptation of Blastocystis to the gut environment [72].

Surprisingly many genes were transferred from bacteria into multicellular eukaryotes. The ancient bacterivorous nematodes acquired cell wall degrading enzymes from several bacterial lineages via HGT [73–75]. The cell wall degrading genes are required for the initial stages in plant pathogenesis, without them plants would be an unavailable niche for the nematode [76]. Therefore, the transfer of those genes allowed the transition of the nematode from a free living state to a plant parasite [77]. Other examples of innovative interdomain HGTs can be found in the tunicates. A cellulose synthase gene (*cesA*) is proposed to have been transferred to the ancestor of the tunicates from a bacterial lineage [78]. Following a gene duplication, CesA1 produces cellulose for the larval tail and CesA2 synthesizes cellulose for the complex filter-feeding house of the ascidians and larvaceans [78]. This HGT played a role in body plan development in tunicates.

Examples of bacteria to animal transfers also reveal the adaptive benefits. The HhMAN1 gene in the coffee berry borer, *Hypothenemus hampei*, was likely transferred from a bacterial lineage [79]. The gene encodes a secreted mannanase that allows the coffee berry borer access the primary seed storage polysaccharide in the coffee plant and ultimately confers an adaptive advantage because *H. hampei* uses the coffee berry as a specific host [79]. The spider mite *Tetranychus urticae* has several genes likely transferred from bacterial lineages; those are genes that encode a secreted fructosidase and a cyanate lyase-encoding gene that may be involved in feeding on cyanogenic plants [80]. These acquisitions have allowed the spider mite to utilize different plants for feeding thereby expanding its host range [80].

The aphid genome, *Acyrthosiphon pisum*, encodes for multiple carotenoids transferred from fungal lineages. These genes allow the aphid to synthesize its own carotenoids rather than to acquire them from food sources as many other animals do [81]. These are only a few of the current examples of interdomain HGTs. As more and more genomes from multicellular organisms become available more interdomain transfers are likely to be revealed.

## 7. Gene Duplication and Gene Transfer

The emergence of new genes from previously noncoding DNA is a rare event (e.g., [82, 83]). Most new genes are believed to originate through gene duplication [84]. In Eukaryotes gene duplications frequently occur in an autochthonous fashion within a single lineage (Figure 1(a)). Mechanisms include tandem, segmental, and chromosomal duplication, retrotransposition, and genome duplications [85]. Of the two genes created, most frequently one accumulates mutations and is no longer maintained under purifying selection and decays [86]. There are two mechanisms by which the duplicated gene can be maintained, subfunctionalization or neofunctionalization. In subfunctionalization, functions of the parent gene are divided among the duplicated genes; in neofunctionalization, after duplication one copy diverges to create a new function. The creation of new functions from duplicated genes appears to be a rare event [87].

Ancient genome duplications have played an important role in vertebrate, plant, and fungi evolution (see [88] for review). In these ancient duplications it is difficult to decide if the whole genome duplication resulted from an autochthonous autopolyploidization or an allopolyploidization following a between-species hybridization (Figure 1(c)). The latter process is particularly important in plant evolution and breeding [89]. Many of these whole-gene duplications are followed by neofunctionalization and subfunctionalizations of various genes throughout the genome. However the above example of the cellulose synthatase genes in the larvacean lineage of tunicates is an example of a gene duplication leading to neofunctionalization in a eukaryote.

The whole-genome duplication of the fungus *Saccharomyces cerevisiae* followed by neofunctionalization of various genes led to the emergence of viral defense mechanisms from translation elongation and the emergence of gene silencing from origin of replication binding proteins [33]. Subfunctionalization events after gene or genome duplications can also arise and create novel regulatory pathways. For example, the maize genome arose from an allotetraploidization between two grass species [90–93]. In the extant maize lineage the *ZAG1* and *ZMM2* genes are necessary for the development of stamens and carpals in the plant. The *ZAG1* gene is expressed throughout carpal development, and the *ZMM2* gene is expressed in maize stamen but not in the immature carpal [94]. It is thought that these genes were expressed in both developing stamens and carpals in the allotetraploid ancestor shortly after the polyploidization event [95]. Over time mutations affecting the regulation of *ZAG1* decrease expression of ZAG1 in stamens but not carpals and mutations affecting the regulation of *ZMM2* eliminated expression in the early carpal but not in stamens [95].

In Bacteria and Archaea autochthonous gene duplications appear to be rare [42, 96]. The typical pathway for gene family extension is through HGT followed by nonhomologous recombination in the recipient. Following the divergence of two lineages, orthologous genes experience substitutions. These might be associated with altered properties of the encoded protein; for example, mutations in an ion translocating subunit of an ATP synthase/ATPase might increase its specificity for protons, thereby changing the specificity for the transported ion from Na^+^ to H^+^ [97], allowing the organism to use the proton motive force for ATP synthesis. When subsequently the two genes end up in the same cell following horizontal gene transfer, they have diverged so much that homologous recombination between the divergent forms is no longer possible (Figure 1(b)). As both genes have different functions, both can be maintained in the recipient through purifying selection. For example, one ATPase might function as ATP synthase driven by a Na^+^ gradient, and the homolog might function in controlling the cellular pH.

## 8. Conclusions

The processes of reticulate evolution lead to innovations and complexity. Horizontal gene transfer whether beneficial or parasitic in nature can lead to innovations and increased complexity. “Beneficial” HGTs provide an immediate selective advantage to the recipient, which increases fitness and guarantees that the transferred gene will be fixed in the recipient's population. Such benefits include but are not limited to innovations in metabolic pathways, expansion of niche adaptations, and in the case of the human gut microbiome can have important secondary implications for the human. “Parasitic” HGTs can also provide innovation, although innovation is more likely to be formed through neutral or nearly neutral pathways to complexity. Gene and genome duplications are another way to spawn innovation and complexity, more so in Eukaryotes than in prokaryotic lineages. In both cases, the horizontal transfer of genetic material and gene and genome duplications are a driving factor in organismal evolution.

## Figures and Tables

**Table 1:** Categories of HGTs leading to innovation and complexity.

Type	“Beneficial” HGTs	“Neutral” HGTs	“Parasitic” HGTs
Definition	HGTs that provide an initial selective advantage to the recipient	HGTs are maintained by random genetic drift	HGTs do not provide an initial selective advantage to the recipient but over time may adapt to have a beneficial function or be maintained via pathways to neutral complexity in the recipient

Examples	(i) Metabolic pathway expansion and invention	(i) Many ORFan genes and genes of limited distribution and with unknown function may be in this category [14, 15]	(i) Inteins (ii) Group I Introns (iii) Group II Introns
	(ii) Adaptation to new ecological niches		
